# Complete chloroplast genome sequence of *Betula alnoides* (Betulaceae)

**DOI:** 10.1080/23802359.2019.1637790

**Published:** 2019-07-12

**Authors:** Mingyu Yin, Junjie Guo, Jie Zeng

**Affiliations:** Research Institute of Tropical Forestry, Chinese Academy of Forestry, Guangzhou, P.R. China

**Keywords:** *Betula alnoides*, chloroplast genome, Betulaceae, phylogenetic analysis

## Abstract

*Betula alnoides* (Betulaceae) is a species widely distributed in north tropical and warm subtropical zones in South-East Asia and southern China. In this study, we described the complete chloroplast genome of *B. alnoides* based on Illumina paired-end sequencing. The chloroplast genome of *B. alnoides* is 160,990 bp long, including two inverted repeats (IRs, 26,022 bp), separated by a large single-copy region (LSC, 89,719 bp) and a small single-copy region (SSC, 19,227 bp). The genome contains 113 genes, including 79 protein-codon genes (PCGs), four rRNA genes, and 30 tRNA genes. The overall GC content of the chloroplast genome was 35.9%. Phylogenetic analysis demonstrated that *B. alnoides* of Sect. *Betulaster* (Spach) Regel was quite different from species of Sect. *Betula*.

## Introduction

*Betula alnoides* Buch. Ham. ex D. Don, a multiple ploidy tree species of the genus *Betula*, is widely distributed in the tropical and warm subtropical regions in South-East Asia and southern China (Zeng et al. 1999). The nuclear *ADH*, ITS and plastid *mat*K sequences have been used in phylogenetic analysis of genus *Betula* (Jarvinen et al. 2004; Wang et al. 2016). However, due to the common hybridization, independent polyploidization, and introgression events (Thórsson et al. 2001; Jarvinen et al. 2004), the phylogenetic relationship between *B. alnoides* and other species in this genus was controversial (Wang et al. 2016). Here, we obtained the complete chloroplast genome sequence of *B. alnoides* which will help us solve this issue.

Leaves of *B. alnoides* were collected from Baoshan (Yunnan, China; 24°49′23″N, 99°56′54″E), and genomic DNA was extracted from silica gel-dried leaves with a modified cetyltrimethylammonium bromide (CTAB) method (Zeng et al., 2002). The DNA was sequenced on Illumina Hiseq4000 Platform (Illumina, San Diego, CA). After we got the next-generation sequencing (NGS) data, the chloroplast genome was assembled with SPAdes and reconfirmed with Geneious (Bankevich et al. 2012; Kearse et al. 2012). Then, the initial annotation was performed on Plann and corrected with Sequin (Huang and Cronk 2015). The complete chloroplast genome sequence of *B. alnoides* with gene annotated was submitted to GenBank (Accession number MK888853).

The chloroplast genome of *B. alnoides* is 160,990 bp in size, comprising a pair of inverted repeats (IRs; 26,022 bp), a LSC region (89,719 bp), and a SSC region (19,227 bp). There are 113 unique genes annotated which including 79 protein-coding genes (PCGs), four ribosomal RNA (rRNA) genes, and 30 transfer RNA (tRNA) genes. Most genes are single-copy genes while 18 genes located in the IRs, including seven PCGs genes, four rRNA genes, and seven tRNA genes. The base composition of the whole chloroplast genome was uneven (31.5% A, 18.3% C, 17.6% G, 32.6% T), with an overall GC content of 35.9%, and the corresponding values of the LSC, SSC, and IRs regions reaching 33.6%, 29.4%, and 42.5%, respectively.

**Figure 1.**
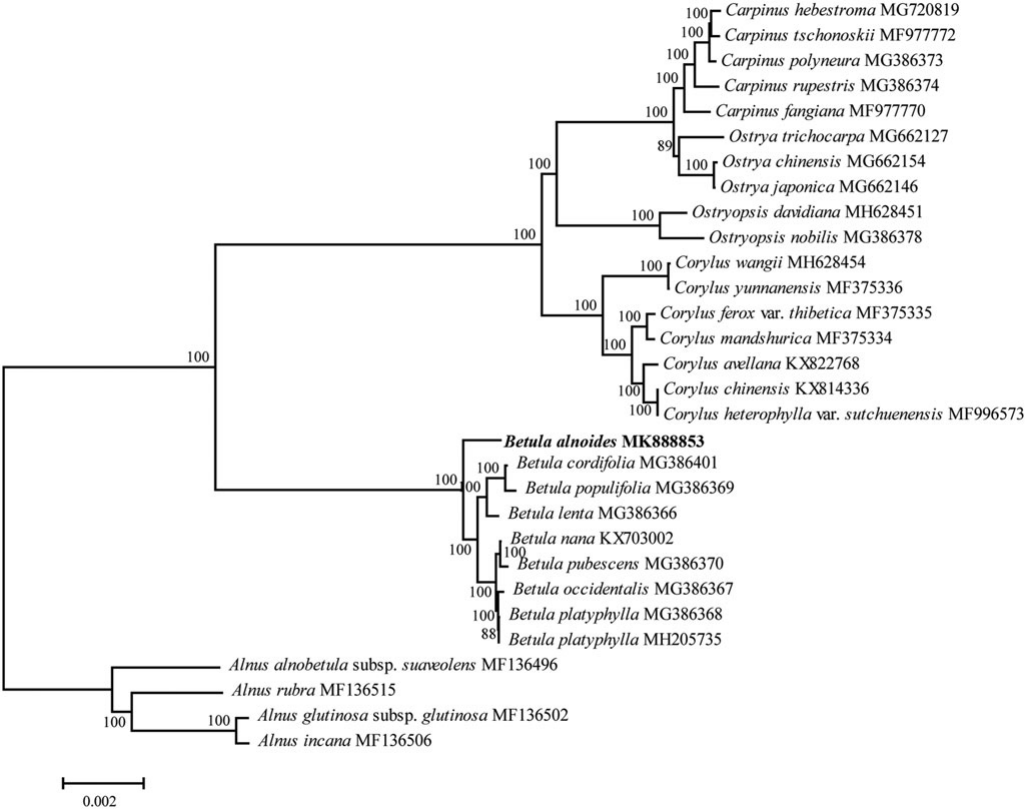
Phylogenetic tree based on chloroplast genomes using the ML method. Ultrafast bootstrap values are shown above the nodes, with 1000 bootstrap replicates. The chloroplast genome sequences of 29 species except for Betula alnoides were downloaded from theNCBI GenBank database (https://www.ncbi.nlm.nih.gov)".

To validate the evolutionary status of *B. alnoides*, we reconstruct the phylogenetic relationships based on the complete chloroplast genomes of eight species from genus *Betula* and 21 complete chloroplast genomes from other genus in Betulaceae. The alignment was performed by software MAFFT (Katoh and Standley 2013). Model Finder (Kalyaanamoorthy et al. 2017) was used for model selection according to the Bayesian information criterion (BIC), and a maximum-likelihood (ML) tree was constructed using IQ-TREE with 1000 boots trap replicates (Nguyen et al. 2015). In this study, we obtained a stable relationship between *B. alnoides* and other species in this genus. It is indicated that *B. alnoides* of Sect. *Betulaster* (Spach) Regel was clearly separated with species of Sect. *Betula* in the genus *Betula*, and it was 100 percent supported. The report of *B. alnoides* complete chloroplast genome will facilitate its further studies in phylogeny, population genetics, genetics resources evaluation, and molecular breeding (Figure 1).
